# Acute kidney injury interacts with *VKORC1* genotype on initiative warfarin dose among heart surgery recipients: a real-world research

**DOI:** 10.1038/s41598-023-46895-2

**Published:** 2023-12-08

**Authors:** Liang Xiong, Feng Yu, Weihong Ge, Hang Xu

**Affiliations:** 1 Department of Pharmacy, Nanjing Drum Tower Hospital, School of Basic Medicine and Clinical Pharmacy, China Pharmaceutical University, Nanjing, China; 2 School of Basic Medicine and Clinical Pharmacy, China Pharmaceutical University, Nanjing, China; 3 Department of Pharmacy, Nanjing Drum Tower Hospital, The Affiliated Hospital of Nanjing University Medical School, Nanjing, China; 4 School of Pharmacy, Faculty of Medicine, Macau University of Science and Technology, Macau SAR, China

**Keywords:** Pharmacogenetics, Interventional cardiology, Epidemiology, Acute kidney injury, Valvular disease, Acute kidney injury

## Abstract

Patients who receive heart valve surgery need anticoagulation prophylaxis to reduce the risk of thrombosis. Warfarin often is a choice but its dosage varies due to gene and clinical factors. We aim to study, among them, if there is an interaction between acute kidney injury and two gene polymorphisms from this study. We extracted data of heart valve surgery recipients from the electronic health record (EHR) system of a medical center. The primary outcome is about the average daily dose of warfarin, measured as an additive interaction effect (INTadd) between acute kidney injury (AKI) and warfarin-related gene polymorphisms. The confounders, including age, sex, body surface area (BSA), comorbidities (i.e., atrial fibrillation [AF], hypertension [HTN], congestive heart failure [CHF]), serum albumin level, warfarin-relevant gene polymorphism (i.e., *CYP2C9*, *VKORC1*), prosthetic valve type (i.e., metal, bio), and warfarin history were controlled via a multivariate-linear regression model. The study included 200 patients, among whom 108 (54.00%) are female. Further, the mean age is 54.45 years, 31 (15.50%) have CHF, and 40 (20.00%) patients were prescribed concomitant amiodarone, which potentially overlays with the warfarin prophylaxis period. During the follow-up, AKI occurred in 30 (15.00%) patients. *VKORC1* mutation (1639G>A) occurred in 25 (12.50%) patients and CYPC29 *2 or *3 mutations presented in 20 patients (10.00%). We found a significant additive interaction effect between AKI and *VKORC1* (− 1.17, 95% CI − 1.82 to − 0.53, p = 0.0004). This result suggests it is probable that there is an interaction between acute kidney injury and the *VKORC1* polymorphism for the warfarin dose during the initial period of anticoagulation prophylaxis.

## Introduction

Patients who underwent heart valve replacement or reparation surgery are required to receive prophylactic anticoagulation^1,2^. Warfarin, which is available in China and has a low cost, is usually the first choice for most patients in our hospital. However, a major drawback of warfarin is the inter-individual heterogeneity in the dosage caused by many factors. One group of factors involves warfarin-related genes^3–7^. Meanwhile, another group of factors is related to demographics and clinical factors^6,8–10^.

Among clinical factors, acute kidney injury is especially of interest because for those who received heart surgery, post-operation acute kidney injury (AKI) is relatively common and is estimated to be as high as 42%, based on a varied definition of cardiac surgery-associated AKI^11–17^. And there are several studies showing AKI has an effect on warfarin dosage. For example, Limdi, N.A. et al. found that moderate and severe kidney impairment was associated with a reduction in the warfarin dose required to maintain the target INR^8^. In another study, Ning et al. found that renal function was correlated with the dose of warfarin^18^. Finally, there are a few other studies concluding kidney function is associated with warfarin response^19,20^.

Because of the relatively common post-operative AKI among heart surgery patients and its correlation with warfarin response. We aim to investigate if there is an interaction between AKI and *CYP2C9*/*VKORC1* polymorphism and its influence on the daily dose of warfarin during hospitalization based.

## Results

Among the 200 patients, 108 (54.00%) are female. Further, the mean age is 54.45 years, and the mean weight of 61.82 kg. Indication as mechanical valve replacement, bio-prosthetic valve replacement, or valve reparation accounts for 35.50%, 41.50%, and 23.00% of patients, respectively. 40 (20.00%) patients were prescribed concomitant amiodarone, which potentially overlays with the warfarin prophylaxis period. AKI occurred in 30 (15.00%) patients. *VKORC1* mutation (1639G>A) occurred in 25 (12.50%) patients and CYPC29 *2 or *3 mutations presented in 20 patients (10.00%). The median days of warfarin prophylaxis until the discharge is 10 days. More patient characteristics are available in Table 1.

**Table 1:** Characteristics of the 200 subjects at the time of enrollment.

Variable	Value
Demographics	
Female sex—no. (%)	108 (54.00)
Age—years (mean ± SD)	55.45 ± 13.23
Age group—no. (%)	
18–29 years	7 (3.50)
30–39 years	18 (9.00)
40–49 years	38 (19.00)
50–59 years	41 (20.50)
60–69 years	72 (36.00)
70–79 years	21 (10.50)
80–89 years	3 (1.50)
Weight, kg (mean ± SD)	61.82 ± 12.25
Weight status—no. (%)*	
Normal	127 (63.50)
Overweight	52 (26.00)
Emaciated	21 (10.50)
Height, cm (mean ± SD)	163.76 ± 8.93
BSA, m^2^ (mean ± SD)	1.67 ± 0.19
BMI, kg/m^2^ (mean ± SD)	22.96 ± 3.55
Current smoker—no. (%)	10 (5.00)
Cardiac function and performance	
Ejection fraction before surgery^¶^ (mean ± SD)	52.52 ± 9.46
Ejection fraction after surgery^¶^ (mean ± SD)	52.51 ± 8.23
Congestive heart failure—no. (%)	31 (15.50)
NYHA of patients with CHF—no. (%)	
Level I	0 (0.00)
Level II	5 (16.13)
Level III	17 (54.84)
Level IV	9 (29.03)
AHA heart failure classification—no. (%)	
Stage A	169 (84.50)
Stage B	0 (0.00)
Stage C	29 (14.50)
Stage D	2 (1.00)
Comorbidities—no. (%)	
Atrial fibrillation (AF)	64 (32.00)
Type 2 diabetes mellitus (T2DM)	12 (6.00)
Stroke	13 (6.50)
Hypertension (HTN)	46 (23.00)
Chronic kidney disease (CKD)	3 (1.50)
Indication for anticoagulant treatment—no. (%)	
Mechanical prosthetic heart valve replacement	71 (35.50)
Bioprosthetic heart valve replacement	83 (41.50)
Heart valve repair	46 (23.00)
Potential concomitant medication interaction—no. (%)	
Amiodarone	40 (20.00)
Low molecular weight heparin (LMWH)	–
Statins	–
Antiplatelets	–
Follow-up—days	
Days of follow-up—no. (%)	
≤ 7 days	44 (22.00)
8–14 days	118 (59.00)
> 14 days	38 (19.00)
Median (days)	10
Interquartile range (days)	8–13
*VKORC1* haplotype	
Non-A/non-A	0 (0.00)
Non-A/A	25 (12.50)
A/A	175 (87.50)
Total	200 (100.00)
*CYP2C9* genotype	
*1/*1	180 (90.90)
*1/*2	–
*1/*3	19 (9.50)
*2/*2	–
*2/*3	–
*3/*3	1 (0.50)
Total	200 (100.00)

*Overweight is defined as a ratio of real body weight to ideal body weight of greater than 1.2; emaciated is defined as a ratio of real body weight to ideal body weight of less than 0.9.

^¶^There is some missing data: 8 patients have preoperative ejection fraction data missing and 6 patients have post-operative ejection fraction data missing.

**Figure 1:**
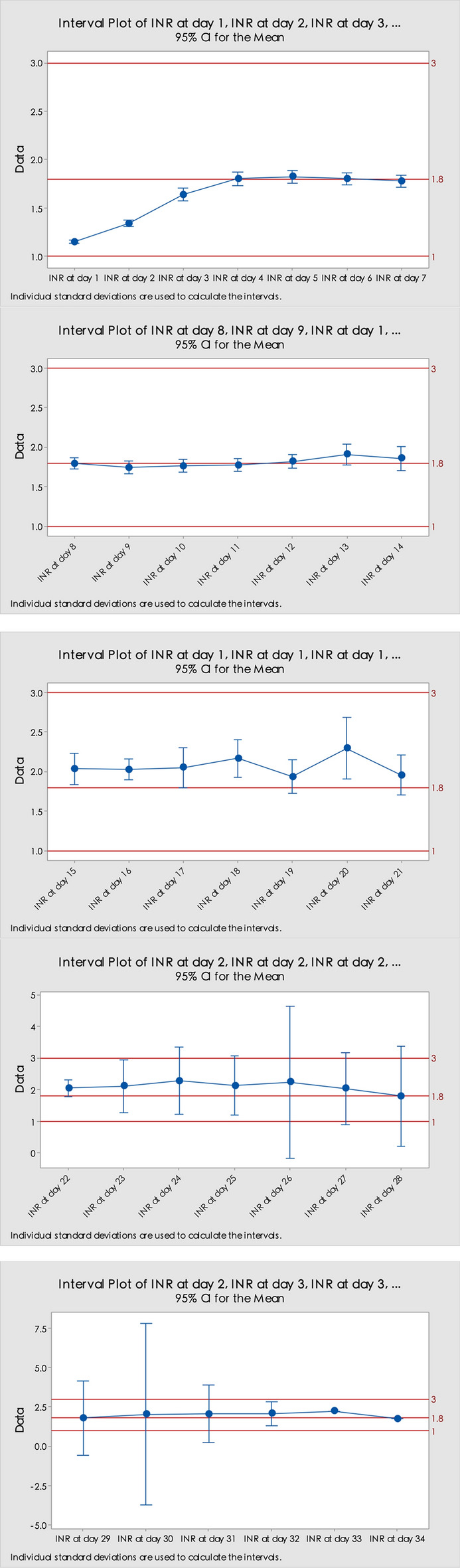
Mean daily INR from enrollment and the confidence interval.

Mean INR values at day 3 (n = 182) and day 7 (n = 167) are 1.64 and 1.78 respectively. 78.50% of patients (n = 157) achieved the lower target INR goal before discharge. The mean average daily dose of warfarin until discharge was 2.68 mg (SD 0.80). Full INR data during hospitalization is summarized in Fig. 1. As shown in Fig. 1, the mean INR value gradually rose and on day 4 it reached the target INR range.

Analysis of data shows the two gene polymorphisms, namely, *CYP2C9* (p = 0.0051) and *VKORC1* (< 0.0001) are independently associated with the average warfarin daily dose. Patients with *CYP2C9* mutations required lower average daily doses, whereas those with *VKORC1* mutation (*VKORC1* 1639G>A) needed higher average daily doses. Clinical characteristics and factors that are independently correlated with the average daily dose are age (p = 0.0005), body surface area (p < 0.001), hypertension (p = 0.0089), CHF (p = 0.0345), and levels of serum albumin (p = 0.0163).

### Primary outcome

Most importantly, a significant interaction between AKI and *VKORC1* (INT_add_ mean − 1.17, 95% CI − 1.82 to − 0.53) was observed (p = 0.0004), and the additive influence of AKI plus *VKORC1* morphism on average daily dose was statistically weaker than the sum of adding each individually. In other words, when acute kidney injury and mutation of *VKORC1* (1639G>A) are both present, their additive net influence on the average daily dose is significantly smaller than the arithmetic sum of the influences of each factor individually. Predicted analysis shows patients who have *VKORC1* non-A genotypes but with acute kidney injury would have a significantly lower mean average daily dose than those without AKI (2.63 mg/day [95% CI 2.02–3.23] vs 3.80 mg/day [3.49–4.12], the value of other model cofactors: male, age 60 years, BSA 1.75 m^2^, no HTN, no CHF, serum albumin 40 g/L, *CYP2C9* wild type). Additional results are presented in Table 2. We did not observe a significant additive interaction between AKI and *CYP2C9*.

**Table 2:** Multi-variable regression result of the 200 subjects.

Variable	Estimate	95% confidence limits	P value
Clinical factors			
Intercept	− 0.22	− 2.23 to 1.80	0.8325
Age	− 0.01	− 0.02 to − 0.01	0.0005
BSA	1.06	0.54 to 1.57	< 0.0001
Hypertension	0.31	0.08 to 0.54	0.0089
CHF	− 0.27	− 0.53 to − 0.02	0.0345
Serum albumin level	0.05	0.01 to 0.09	0.0163
Genetic factors			
* CYP2C9*	− 0.43	− 0.73 to − 0.13	0.0051
* VKORC1*	1.07	0.78 to 1.37	< 0.0001
Interaction			
VKORC*AKI	− 1.17	− 1.82 to − 0.53	0.0004

In this study, we did not include a sensitivity analysis for amiodarone because this would cause a significant sample loss of whom the AKI or gene polymorphism was present. In addition, the majority of patients who took amiodarone stopped this drug within 4 days. Finally, our multi-variable linear regression model does include drug-to-drug interaction due to amiodarone as a confounder and we did not observe a significant association between it and the dependent variable. Of note, both drug labels indeed warn there is a drug interaction between amiodarone and warfarin and our study does not conflict with this fact because we think: (1) the sample size of this study has limited the power of the statistical regression model, (2) the majority of patients who took amiodarone stopped this drug within 4 days, which possibly did not reach sufficient interaction strengthen for us to successfully observe the difference due to limited statistical power.

## Discussion

The primary discovery of our study is that we observed a significant additive interaction between the occurrence of post-operation acute kidney injury and *VKORC1* polymorphism. The additive interaction effect for average daily dose difference is negative, which means the additive effect on average dose is smaller when both AKI and 1639G>A were present, compared with the sum of both effects being added individually. A secondary finding is that the influence of the *VKORC1* genotype on the average daily warfarin dose depended on the cofactor AKI after surgery. The presence of postoperative AKI significantly extenuated the impact of *VKORC1* mutation (1639G>A) on the average daily dose. To explain these observations, we base our postulations on pharmacokinetics and pharmacodynamics.

According to the definition of additive interaction effect, the components of INT_add_, i.e. (Dose_11_ − Dose_00_) – [(Dose_10_ – Dose_00_) + (Dose_01_ – Dose_00_)], could be redressed and then expressed by the function of pharmacokinetic parameters clearance, unbound target drug concentration, and unbound fraction. Here we define clearance and unbound target drug plasma concentration under different cases as follows [Eq. (1)]:

1 $$Cl_{c} \int {\frac{{C_{GA,f} }}{{f_{u,AKI} }}} dt - Cl\int {\frac{{C_{f} }}{{f_{u} }}} dt - \left[ {\left( {Cl_{c} \int {\frac{{C_{f} }}{{f_{u,AKI} }}} dt - Cl\int {\frac{{C_{f} }}{{f_{u} }}} dt} \right) + \left( {Cl\int {\frac{{C_{GA,f} }}{{f_{u} }}} dt - Cl\int {\frac{{C_{f} }}{{f_{u} }}} dt} \right)} \right]$$

Equation (1) is re-expressed into Eq. (2) by some algebras. The math process is shown below,

$$\begin{gathered} Cl_{c} \int {\frac{{C_{GA,f} }}{{f_{u,AKI} }}} dt - Cl\int {\frac{{C_{f} }}{{f_{u} }}} dt - \left[ {\left( {Cl_{c} \int {\frac{{C_{f} }}{{f_{u,AKI} }}} dt - Cl\int {\frac{{C_{f} }}{{f_{u} }}} dt} \right) + \left( {Cl\int {\frac{{C_{GA,f} }}{{f_{u} }}} dt - Cl\int {\frac{{C_{f} }}{{f_{u} }}} dt} \right)} \right] \hfill \\ \downarrow \hfill \\ Cl_{c} \int {\frac{{C_{GA,f} }}{{f_{u,AKI} }}} dt - Cl\int {\frac{{C_{f} }}{{f_{u} }}} dt - Cl_{c} \int {\frac{{C_{f} }}{{f_{u,AKI} }}} dt + Cl\int {\frac{{C_{f} }}{{f_{u} }}} dt - Cl\int {\frac{{C_{GA,f} }}{{f_{u} }}} dt + Cl\int {\frac{{C_{f} }}{{f_{u} }}} dt \hfill \\ \downarrow \hfill \\ Cl_{c} \int {\frac{{C_{GA,f} }}{{f_{u,AKI} }}} dt - Cl_{c} \int {\frac{{C_{f} }}{{f_{u,AKI} }}} dt - \left( {Cl\int {\frac{{C_{GA,f} }}{{f_{u} }}} dt + Cl\int {\frac{{C_{f} }}{{f_{u} }}} dt} \right) \hfill \\ \downarrow \hfill \\ \frac{{Cl_{c} }}{{f_{u,AKI} }}\left( {\int {C_{GA,f} } dt - \int {C_{f} dt} } \right) - \frac{Cl}{{f_{u} }}\left( {\int {C_{GA,f} } dt - \int {C_{f} dt} } \right) \hfill \\ \downarrow \hfill \\ \left( {\frac{{Cl_{c} }}{{f_{u,AKI} }} - \frac{Cl}{{f_{u} }}} \right)\left( {\int {C_{GA,f} } dt - \int {C_{f} dt} } \right) \hfill \\ \downarrow \hfill \\ \left( {\int {C_{GA,f} } dt - \int {C_{f} dt} } \right)\left( {\frac{{Cl_{c} }}{{f_{u,AKI} }} - \frac{Cl}{{f_{u} }}} \right) \hfill \\ \end{gathered}$$

Which equals to below [Eq. (2)],

2 $$\left( {\int {C_{GA,f} } dt - \int {C_{f} dt} } \right)\left( {\frac{{Cl_{c} }}{{f_{u,AKI} }} - \frac{Cl}{{f_{u} }}} \right)$$

Cl_c_: compromised drug clearance due to AKI.

Cl: normal drug clearance when no AKI present.

C_GA, f_: the target unbound drug concentration required for *VKORC1* 1639G > A (GA) patients.

C_f_: the target unbound drug concentration required for *VKORC1* AA patients.

f_u, AKI_: unbound drug fraction under the condition of AKI.

f_u_: unbound drug fraction in normal condition.

Because the hepatic clearance of warfarin is a capacity-limited process (low hepatic extraction ratio), the hepatic clearance of warfarin could be rounded to the function of the intrinsic hepatic clearance timing the unbound fraction of warfarin^21^. Finally, the INT_add_ could be expressed by the formula below [Eq. (3)],

3 $$\left( {\int {C_{GA,f} } dt - \int {C_{f} dt} } \right)\left( {Cl_{{{\text{int}} ,c}} - Cl_{{\text{int}}} } \right)$$

Cl_int,c_: compromised intrinsic clearance due to AKI.

Cl_int_: intrinsic clearance in normal condition.

In theory, we assume the relationship between Cl_int,c_ and Cl_int_ is Cl_int,c_ < Cl_int_, where the smaller value of Cl_int,c_ was postulated to be the result of compromised hepatic metabolism along with some cases of AKI. There are some pieces of evidence supporting this assumption^22–26^. Meanwhile, evidence indirectly suggests that patients who have the *VKORC1* GA genotype require a higher target unbound drug concentration than patients with the AA genotype, which means C_GA,f_ > C_f_^27–30^. With the above possible quantitative relationship among intrinsic clearances with and without AKI, and in addition to the probable quantitative relationship between target free drug concentrations C_GA,f_ and C_f_, the INT_add_ expressed as Eq. (3) predicts a negative value. However, attention is needed that the daily warfarin doses that we recorded and used to estimate the INTadd were the dose during the initial stage of anticoagulation instead of the steady state dose. Although it is the steady-state dose of warfarin that correlates with the anticoagulation response, the dose records we use in this study were tightly adjusted based on the INR response during the initial stage after surgery. Figure 1 shows the average INR of our samples during the follow-up and on an average level it rose and achieved the target INR value on day 4 and stayed in the target INR range for most of the time thereafter. Therefore, the dose we recorded probably is a reasonable substitution for the steady-state dose of warfarin.

We did not observe a significant INT_add_ related to AKI and *CYP2C9* polymorphism. Similarly, we hypothesize the explanation for this result based on clinical pharmacology. Using the very similar method as above, we express the INT_add_ as Eq. (4) below.

4 $$Cl_{2c9,AKI} \int {\frac{{C_{f} }}{{f_{u,AKI} }}} dt - Cl\int {\frac{{C_{f} }}{{f_{u} }}} dt - \left( {Cl_{c,AKI} \int {\frac{{C_{f} }}{{f_{u,AKI} }}} dt - Cl\int {\frac{{C_{f} }}{{f_{u} }}} dt + Cl_{2c9} \int {\frac{{C_{f} }}{{f_{u} }}} dt - Cl\int {\frac{{C_{f} }}{{f_{u} }}} dt} \right)$$

After several algebras, the INT_add_ involving AKI and *CYP2C9* finally is expressed as Eq. (5).

5 $$\left( {Cl_{{{\text{int}} ,2c9,AKI}} - Cl_{{{\text{int}} ,2c9}} } \right)\int {C_{f} dt - \left( {Cl_{{{\text{int}} ,c}} - Cl_{{\text{int}}} } \right)} \int {C_{f} dt}$$

Cl_int,2c9,AKI_: compromised intrinsic clearance due to AKI of *CYP2C9* polymorphism carriers.

Cl_int,2c9_: intrinsic clearance in normal condition among *CYP2C9* polymorphism carriers.

Cl_int,c_: compromised intrinsic clearance due to AKI in *CYP2C9* wild genotype individuals.

Cl_int_: intrinsic clearance in normal condition among *CYP2C9* wild genotype individuals.

As Eq. (5) shows, the INTadd of AKI and *CYP2C9* is a function of the difference of differences. This exactly means the difference between the AKI-induced intrinsic drug clearance differences (reductions) for patients with and without *CYP2C9* polymorphism determines the direction and values of INTadd (AKI and *CYP2C9*). Therefore, for patients with and without *CYP2C9* polymorphism, the relative reductions of the intrinsic drug clearance due to AKI are, $\frac{{Cl_{{{\text{int}} ,2c9,AKI}} - Cl_{{{\text{int}} ,2c9}} }}{{Cl_{{{\text{int}} ,2c9}} }}$ and $\frac{{Cl_{{{\text{int}} ,c}} - Cl_{{\text{int}}} }}{{Cl_{{\text{int}}} }}$, respectively.

We postulate that the degree of the impact of AKI on intrinsic clearance is different for different *CYP2C9* genotypes. More precisely, because Cl_int,2c9_, is quantitively smaller than Cl_int_, we think that the effect of AKI on the intrinsic clearance is more profound for *CYP2C9* polymorphism carriers than for *CYP2C9* wild genotype individuals. This different degree of impact of AKI on the intrinsic clearance possibly results in quantitively similar absolute reduction values for individuals with and without *CYP2C9* polymorphism, respectively (i.e., individuals with *CYP2C9* polymorphism are more sensitive to AKI’s impact on intrinsic clearance). This is our postulated explanation for our observation about INTadd between *CYP2C9* polymorphism and AKI.

To explain the second observation that the influence of mutation of *VKORC1* genotype (1639G>A) on average daily warfarin dose depended on the presence or absence of AKI after surgery, we also based our elaboration on pharmacokinetics and pharmacodynamics. First, warfarin has an antagonistic effect on vitamin K epoxide reductase. Second, the dose–effect curve (log-scale) shifts to the right when *VKORC1* gene mutation (1639G>A) presents due to less responsiveness to warfarin, substantiated by two recent studies^31,32^. This right shift makes patients with *VKORC1* genotype GG/GA (1639G>A) less responsive to warfarin, which requires a higher drug concentration to achieve the same therapeutic drug response. In the same words, patients with *VKORC1* AA genotype require significantly lower therapeutic target drug concentration than those with the 1639G>A GG/GA genotype.

In the case of AKI, we postulate the metabolic function of hepatocytes was negatively affected or even inhibited. This was supported by the research by Dixon et al.^35,36^. For drugs with a low hepatic extraction ratio such as warfarin, drug hepatic clearance is approximately the direct result of the hepatic intrinsic clearance for that drug. This is reflected by Rowland’s Equation^33^ where for warfarin, Cl(h) = f × Cl_int_. Because the hepatic intrinsic clearance is a pure measure of the ability of the liver enzymes to metabolize a drug, in the case of negatively changed hepatocyte metabolic function from AKI, we think this reduced enzyme metabolic function probably means a reduced intrinsic hepatic clearance for warfarin. Therefore, for warfarin, the final result is a reduced hepatic clearance, and probably this potents patients to increased warfarin exposure. As a result, in patients with *VKORC1* genotype GG/GA and who had AKI, it requires lower daily doses, which explains our secondary observation. Below is the Rowland’s Equation.

$${\text{Hepatic}}\;{\text{Clearance}}:\;{\text{Cl}}({\text{h}}) = {\text{Q}}\left[ {\left( {{\text{f}} \times {\text{Cl}}_{{\text{int}}} } \right)/\left( {{\text{Q}} + {\text{f}} \times {\text{Cl}}_{{\text{int}}} } \right)} \right]$$

Q = hepatic blood flow, f = fraction of free drug (not bound), Cl_int_ = intrinsic capacity of the hepatocytes to metabolize a drug.

Our study has several limitations. First, the daily warfarin doses that we recorded were during the initial stage of anticoagulation instead of during the steady state period. However, our healthcare providers monitor the INR and adjust the dosage accordingly in a timely fashion. Therefore, the doses we recorded were associated with the steady-state dosage. Second, we did not conduct multicenter research and the sample size is relatively small, which restricted the statistical power of this study. Third, although we used multi-variable regression to adjust confounders, unknown cofactors might exist and not be adjusted. Most importantly, our study provides a piece of evidence that genetic and clinical factors might interact to alter the drug response of patients taking warfarin.

In conclusion, our study provides evidence that acute kidney injury possibly interacted with *VKORC1* polymorphism and this interaction significantly affected the dose of warfarin. For anticoagulation through warfarin, genetic and clinical factors of patients might interact and healthcare providers should consider these potential interactions when titrating the dose of warfarin.

## Methods

**Figure 2:**
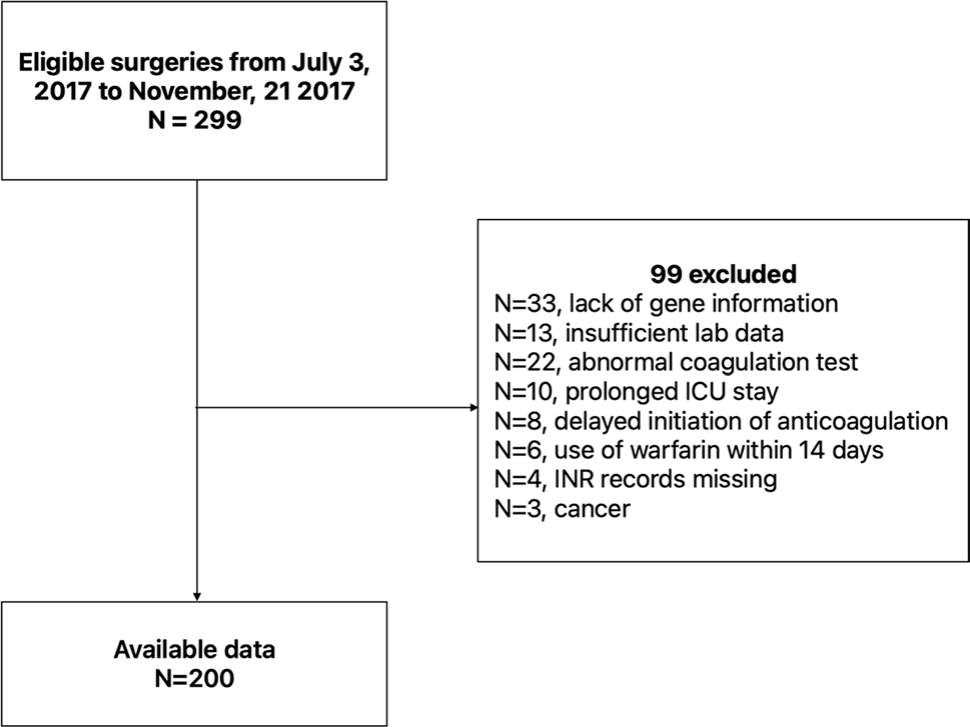
Study flow diagram.

This study was a single-center cohort study and it was approved by the local institutional review board and an institutional research ethics committee of Nanjing Drum Tower Hospital. All methods were performed in accordance with the relevant guidelines and regulations and informed consent was obtained from all subjects and/or their legal guardian(s). From July 2017 to November 2017, we consecutively collected data from 200 patients who underwent heart valve replacement or repair surgery in the Department of Cardiothoracic Surgery, Nanjing Drum Tower Hospital. Patient demographics, laboratory test results, drug order records, and genetic test results were extracted from the electronic health record system and used for analysis. The STROBE flowchart of this study is presented in Fig. 2.

The primary outcome is the additive interaction effect (INT_add_) for average daily dose difference which is defined as INT_add_ = (Dose_11_ − Dose_00_) − [(Dose_10_ − Dose_00_) + (Dose_01_ − Dose_00_)], where Dose in Dose_xy_ is the average daily dose of warfarin during hospitalization following surgery and subscripts x and y serially designate the presence or absence of AKI and warfarin-related gene polymorphism (i.e., *CYP2C9* or *VKORC1*) respectively, where the value of 1 means presence and 0 absence of corresponding factors^34^. For example, the dose of individuals who neither have AKI nor gene polymorphism is designated as Dose_00_, whereas the dose of those who have both conditions is Dose_11_, and the dose of those with either AKI or gene polymorphism is designates as Dose_10_ or Dose_01_, respectively. The INT_add_ could be quantitatively estimated directly from the coefficients of a multiple linear regression model. If the confidence intervals of INT_add_ do not cross zero, we would conclude there is a significant additive interaction exists. AKI is defined according to the criteria: a ≥ 50% increase of serum creatinine within 7 days or ≥ 0.3 mg/dL within 3 days, or a urine output below 400 mL per day.

Categorical variables were calculated as numbers and percentages, and continuous variables were calculated as the means and SD. We constructed a multiple linear regression model to make the statistical inference. The dependent variable is the average daily dose of warfarin meanwhile independent possible confounders contain age, sex, BSA, comorbidities (i.e., AF, hypertension, CHF), hypoalbuminemia, drug-to-drug interaction due to amiodarone, warfarin-related gene polymorphism (i.e., *CYP2C9*, *VKORC1*), and prosthetic valve type (i.e., metal, bio), and warfarin history. AKI is the independent variable of this model and two interaction terms (i.e., between AKI and *CYP2C9*, and AKI and *VKORC1*) were added to this regression model, and their regression coefficients represent the INT_add_ for the corresponding interaction, respectively. Type I statistic error limit is set to 5% and all confidence intervals are set to 95%. All statistical analyses were performed using SAS OnDemand for Academics.

## Data Availability

The data underlying this article will be shared on reasonable request to the corresponding author.

## References

[1] Vahanian A (2021). 2021 ESC/EACTS guidelines for the management of valvular heart disease. Developed by the task force for the management of valvular heart disease of the European Society of Cardiology (ESC) and the European Association for Cardio-Thoracic Surgery (EACTS). Eur. Heart J..

[2] Otto CM (2021). 2020 ACC/aha guideline for the management of patients with valvular heart disease: A report of the American College of Cardiology/American Heart Association Joint Committee on Clinical Practice Guidelines. Circulation.

[3] Fung E (2012). Effect of genetic variants, especially CYP2C9 and VKORC1, on the pharmacology of warfarin. Semin. Thromb. Hemost..

[4] Zhang J, Chen Z, Chen C (2016). Impact of CYP2C9, VKORC1 and CYP4F2 genetic polymorphisms on maintenance warfarin dosage in Han-Chinese patients: A systematic review and meta-analysis. Meta Gene.

[5] Hillman MA (2004). Relative impact of covariates in prescribing warfarin according to CYP2C9 genotype. Pharmacogenetics.

[6] Sconce EA (2005). The impact of CYP2C9 and VKORC1 genetic polymorphism and patient characteristics upon warfarin dose requirements: Proposal for a new dosing regimen. Blood.

[7] Aquilante CL (2006). Influence of coagulation factor, vitamin K epoxide reductase complex subunit 1, and cytochrome P450 2C9 gene polymorphisms on warfarin dose requirements. Clin. Pharmacol. Ther..

[8] Limdi NA (2010). Warfarin dosing in patients with impaired kidney function. Am. J. Kidney Dis..

[9] Ather S (2016). Effect of left ventricular systolic dysfunction on response to warfarin. Am. J. Cardiol..

[10] Gurwitz JH, Avorn J, Ross-Degnan D, Choodnovskiy I, Ansell J (1992). Aging and the anticoagulant response to warfarin therapy. Ann. Intern. Med..

[11] Wang Y, Bellomo R (2017). Cardiac surgery-associated acute kidney injury: Risk factors, pathophysiology and treatment. Nat. Rev. Nephrol..

[12] Carrascal Y, Laguna G, Blanco M, Pañeda L, Segura B (2021). Acute kidney injury after heart valve surgery in elderly patients: Any risk factors to modify?. Braz. J. Cardiovasc. Surg..

[13] Al-Githmi IS (2022). Acute kidney injury after open heart surgery. Cureus.

[14] Djordjević A, Šušak S, Velicki L, Antonič M (2021). Acute kidney injury after open-heart surgery procedures. Acta Clin. Croat..

[15] Serraino GF (2021). Risk factors for acute kidney injury and mortality in high risk patients undergoing cardiac surgery. PLoS ONE.

[16] Helgason D (2016). Acute kidney injury and outcome following aortic valve replacement for aortic stenosis. Interact. Cardiovasc. Ther..

[17] Ryugo M (2020). Risk analysis of acute kidney injury after cardiac surgery and protective effect by less invasive surgery. Kyobu Geka Jpn. J. Thorac. Surg..

[18] Ning X (2021). Influence of renal insufficiency on anticoagulant effects and safety of warfarin in Chinese patients: Analysis from a randomized controlled trial. Naunyn-schmiedeb. Arch. Pharmacol..

[19] Limdi MA, Crowley MR, Beasley TM, Limdi NA, Allon M (2013). Influence of kidney function on risk of hemorrhage among patients taking warfarin: A cohort study. Am. J. Kidney Dis..

[20] Limdi NA (2015). Influence of kidney function on risk of supratherapeutic international normalized ratio-related hemorrhage in warfarin users: A prospective cohort study. Am. J. Kidney Dis..

[21] Mehvar R (2018). Clearance concepts: Fundamentals and application to pharmacokinetic behavior of drugs. J. Pharm. Pharm. Sci..

[22] Vilay AM, Churchwell MD, Mueller BA (2008). Clinical review: Drug metabolism and nonrenal clearance in acute kidney injury. Crit. Care.

[23] Touchette MA, Slaughter RL (1991). The effect of renal failure on hepatic drug clearance. Ann. Pharmacother..

[24] Elston AC, Bayliss MK, Park GR (1993). Effect of renal failure on drug metabolism by the liver. Br. J. Anaesth..

[25] Sun H, Frassetto L, Benet LZ (2006). Effects of renal failure on drug transport and metabolism. Pharmacol. Ther..

[26] Nolin T, Naud J, Leblond F, Pichette V (2008). Emerging evidence of the impact of kidney disease on drug metabolism and transport. Clin. Pharmacol. Ther..

[27] Li J (2018). Impact of VKORC1, CYP4F2 and NQO1 gene variants on warfarin dose requirement in Han Chinese patients with catheter ablation for atrial fibrillation. BMC Cardiovasc. Disord..

[28] Chertovskikh YV, Malova EU, Maksimova NR, Popova NV, Sychev DA (2015). VKORC1 polymorphisms and warfarin maintenance dose in population of Sakha (Yakuts). Int. J. Risk Saf. Med..

[29] Ye C (2013). Variability of warfarin dose response associated with CYP2C9 and VKORC1 gene polymorphisms in Chinese patients. J. Int. Med. Res..

[30] Natarajan S (2013). Effect of CYP2C9 and VKORC1 genetic variations on warfarin dose requirements in Indian patients. Pharmacol. Rep..

[31] Li S, Liu S, Liu XR, Zhang MM, Li W (2020). Competitive tight-binding inhibition of VKORC1 underlies warfarin dosage variation and antidotal efficacy. Blood Adv..

[32] Wu S (2018). Warfarin and vitamin K epoxide reductase: A molecular accounting for observed inhibition. Blood.

[33] Rowland M, Tozer TN, Rowland M, Tozer TN (1995). Clinical Pharmacokinetics. Concepts and Applications.

[34] Vandenbroucke JP (2007). Strengthening the reporting of observational studies in epidemiology (STROBE): Explanation and elaboration. PLoS Med..

[35] Dixon J, Lane K, MacPhee I, Philips B (2014). Xenobiotic metabolism: The effect of acute kidney injury on non-renal drug clearance and hepatic drug metabolism. Int. J. Mol. Sci..

[36] Philips BJ, Lane K, Dixon J, MacPhee I (2014). The effects of acute renal failure on drug metabolism. Expert Opin. Drug Metab. Toxicol..

